# Cecal Microbiota of Free-Range Hens Varied With Different Rearing Enrichments and Ranging Patterns

**DOI:** 10.3389/fmicb.2021.797396

**Published:** 2022-02-11

**Authors:** Md Saiful Bari, Sarbast K. Kheravii, Yadav S. Bajagai, Shu-Biao Wu, Chake Keerqin, Dana L. M. Campbell

**Affiliations:** ^1^School of Environmental and Rural Science, University of New England, Armidale, NSW, Australia; ^2^Agriculture and Food, Commonwealth Scientific and Industrial Research Organisation (CSIRO), Armidale, NSW, Australia; ^3^Department of Dairy and Poultry Science, Chattogram Veterinary and Animal Sciences University, Chattogram, Bangladesh; ^4^Institute for Future Farming Systems, Central Queensland University, Rockhampton, QLD, Australia

**Keywords:** bacteria, gut, indoor, outdoor, 16S rRNA, novel objects, perching structures, RFID

## Abstract

Free-range pullets are reared indoors but the adult hens can go outside which is a mismatch that may reduce adaptation in the laying environment. Rearing enrichments might enhance pullet development and adaptations to subsequent free-range housing with impact on behavior and health measures including gut microbiota. Adult free-range hens vary in range use which may also be associated with microbiota composition. A total of 1,700 Hy-Line Brown^®^ chicks were reared indoors across 16 weeks with three enrichment treatment groups: “control” with standard litter housing, “novelty” with weekly changed novel objects, and “structural” with custom-designed perching structures in the pens. At 15 weeks, 45 pullet cecal contents were sampled before moving 1,386 pullets to the free-range housing system. At 25 weeks, range access commenced, and movements were tracked via radio-frequency identification technology. At 65 weeks, 91 hens were selected based on range use patterns (“indoor”: no ranging; “high outdoor”: daily ranging) across all rearing enrichment groups and cecal contents were collected for microbiota analysis via 16S rRNA amplicon sequencing at V3-V4 regions. The most common bacteria in pullets were unclassified Barnesiellaceae, *Prevotella, Blautia* and *Clostridium* and in hens Unclassified, *Ruminococcus*, unclassified Lachnospiraceae, unclassified Bacteroidales, unclassified Paraprevotellaceae YRC22, and *Blautia*. The microbial alpha diversity was not significant within the enrichment/ranging groups (pullets: *P* ≥ 0.17, hen rearing enrichment groups: *P* ≥ 0.06, hen ranging groups: *P* ≥ 0.54), but beta diversity significantly varied between these groups (pullets: *P* ≤ 0.002, hen rearing enrichment groups: *P* ≤ 0.001, hen ranging groups: *P* ≤ 0.008). Among the short-chain fatty acids (SCFAs), the propionic acid content was higher (*P* = 0.03) in the novelty group of pullets than the control group. There were no other significant differences in the SCFA contents between the rearing enrichment groups (all *P* ≥ 0.10), and the ranging groups (all *P* ≥ 0.17). Most of the genera identified were more abundant in the indoor than high outdoor hens. Overall, rearing enrichments affected the cecal microbiota diversity of both pullets and adult hens and was able to distinguish hens that remained inside compared with hens that ranging daily for several hours.

## Introduction

Numerous species of bacteria are sheltered in the gastro-intestinal (GI) tract of chickens, known as gut microbiota (Rychlik, 2020; Madlala et al., 2021), which play an important role in physiological processes including digestion, absorption, health, and production (Pourabedin and Zhao, 2015). Additionally, gut microbiota provide health benefits through inhibiting chronic diseases (Bavananthasivam et al., 2021), defending the host from various pathogens (Waite and Taylor, 2014), and enhancing gut health (Diaz Carrasco et al., 2019). Gut microbiota also contribute to pathogen expulsion from the host and development of the host immune system (Yeoman et al., 2012; Pan and Yu, 2014). Within chickens, distinct microbial communities are present across different areas of the GI tract with the complexity and absolute counts being comparatively high in the cecum (Rychlik, 2020). Fu et al. (2018) explored the gut microbiota diversity of laying hens based on 16S rRNA sequencing in China and found the Bacterioidetes, then Firmicutes phyla were the most predominant among the cecal microbes. Actinobacteria and Proteobacteria are also typically represented in the ceca of most adult chickens although there can be high variation in microbial composition between individuals (Rychlik, 2020). The most common genera of microbiota in the ceca of laying hens have been shown to be the *Bacteroides* and *Prevotella* (Yan et al., 2017). Identifying the composition of microbes within chickens is important for understanding their function and how they may be affected by internal and external environmental parameters.

Research to date in chickens (both meat birds and egg laying hens) has shown that gut microbiota varied across different ages and production stages of both broilers (Han et al., 2016; Jurburg et al., 2019) and laying hens (Videnska et al., 2014; Ngunjiri et al., 2019; Joat et al., 2021). Microbiota populations, their diversity, and composition varied in hens with different diet compositions (Borrelli et al., 2017; Zhou et al., 2021). There is also some evidence that microbiota composition can vary among groups of hens selected for behavioral differences in feather pecking (van der Eijk et al., 2019), although other studies have not found significant differences (Borda-Molina et al., 2021). Housing environments can affect microbial composition with differences found between broiler chickens that had fresh or reused litter on the floor (Wang et al., 2016), or different types of litter substrates (Torok et al., 2009). Several studies comparing different types of indoor cage and cage-free systems (Nordentoft et al., 2011; Adhikari et al., 2020) or caged indoor and outdoor systems in laying hens (Cui et al., 2017; Chen et al., 2019; Hubert et al., 2019; Schreuder et al., 2020, 2021; Seidlerova et al., 2020) have highlighted the impact that variation in the housing environment might have on the gut microbiota diversity and populations.

To date, some studies on housing system impacts in laying hens have made comparisons between hens that are housed inside (caged or cage-free systems), or housed cage-free with access to an outdoor area in both experimental (Chen et al., 2019; Hubert et al., 2019) and commercial settings (Cui et al., 2017; Schreuder et al., 2020, 2021; Seidlerova et al., 2020). Results across these studies have shown that hens kept in backyard flocks outside or given outdoor range access had lower diversity of bacterial species than indoor-housed hens (cloacal microbiota: (Schreuder et al., 2020); cecal microbiota: Seidlerova et al. (2020). Alternatively, other studies have found greater intestinal/cecal microbial diversity in the hens with outdoor access (Cui et al., 2017; Chen et al., 2019; Hubert et al., 2019), and Schreuder et al. (2021) found no substantial differences in cloacal microbiota between hens with indoor only or outdoor access. Adult hens within a free-range system have access to an outdoor area, but not all hens will make use of this resource with some hens ranging infrequently or not at all while others range daily for several hours (Campbell et al., 2020a). Previous research has determined this variation in ranging patterns correlates with variation in fear behavior and other welfare measures (Campbell et al., 2020a). Thus, individual variation in gut microbiota may also be expected. To date, research investigating this is limited (Ruhnke et al., 2018).

Free-range egg production systems in Australia have been increasing to meet consumer preferences for both perceived better-quality eggs and perceived benefits to hen welfare (Bray and Ankeny, 2017; Scrinis et al., 2017). However, free-range housing can have both positive and negative impacts on hen welfare (Campbell et al., 2020a) which may in part be dependent on the discrepancies between rearing and adult housing (Janczak and Riber, 2015). In Australia and elsewhere internationally, free-range pullets are reared inside, but the adult hens have outdoor access. As outdoor access for pullets is challenging due to vaccination requirements and rearing shed construction, enriching the rearing environment instead may improve adaptation of the adult hens. These differences in rearing environments can affect bird behavioral, physical, and physiological development (Campbell et al., 2019) and may also affect the microbiota composition and diversity of the pullets as they grow.

The data presented in this study were from birds that were part of a larger study on the effects of rearing enrichments and ranging variation on the behavior, health, welfare, and production of free-range laying hens (Bari et al., 2020a,b,c, 2021; Campbell et al., 2020c,b). The results across the wider project showed that rearing enrichments had few effects on the behavior and welfare of pullets (Campbell et al., 2020c), but there were impacts of rearing enrichments across the flock cycle in the adult birds. For example, the enriched-reared hens had better plumage coverage (Bari et al., 2020b,c), increased ranging (Campbell et al., 2020b), and hens that ranged for longer had lower body weight but better plumage (Bari et al., 2020c). Thus, if enrichment in rearing had multiple effects on the development of the pullets, including long-term impacts, enrichment might also affect the microbiota of both pullets and adult hens. Individual variation in ranging behavior has previously been correlated with other physical and behavioral differences and, thus, may also be correlated with differences in microbiota composition in adult hens. This study was conducted to evaluate the gut microbiota composition and diversity of the pullets from different rearing environments, and the microbiota composition and diversity of hens from different rearing enrichment groups and ranging patterns. This study represents a first step toward understanding the impacts that rearing environments and individual variation in ranging can have on internal bacterial communities. Short-chain fatty acids were also assessed to support any differences in microbiota although the functional link between microbiota and bird health, welfare, or production were not assessed. A more diverse microbiota population was predicted in the enriched pullets and adult hens than the control groups, but the direction of differences in cecal microbiota diversity between the indoor hens and outdoor rangers was uncertain.

## Materials and Methods

### Ethical Approval

The University of New England Animal Ethics Committee approved all experimental procedures (AEC17-092).

### Pullet Housing

A flock of 1,700 commercially supplied Hy-Line^®^ Brown chicks were reared indoors at the Rob Cumming Poultry Innovation Centre at the University of New England, Armidale, NSW, Australia. The day-old chicks were reared across 16 weeks within 9 pens (6.2 mL × 3.2 mW) distributed across three separate rooms with 3 separate rearing enrichment treatments as previously described (Bari et al., 2020a,b,c). The rearing treatments included a control group with rice hulls as floor litter only, a novelty group where novel objects were also added and changed at weekly intervals (e.g., balls, bottles, bricks, brooms, brushes, buckets, containers, pet toys, plastic pipes etc.) and a structural group where five custom-designed H-shaped perching structures (L, W, H = 0.60 m) with two solid panels and one open-framed side were placed for the whole period of rearing. The pullets were isolated visually but not acoustically from the pullets of other pens via shade cloth on the wire pen dividers. At 16 weeks of age, bird density was approximately 15 kg/m^2^ or 9 pullets/m^2^ (average 174–190 pullets/pen).

The pullets were provided with *ad libitum* access to feed (commercially formulated mash) and water using round feeders and nipple drinker lines. The resources provided met or exceeded the current Australian Model Code of Practice for the Welfare of Animals Domestic Poultry (Primary Industries Standing Committee, 2002). Artificial lighting and temperature schedules followed the recommended Hy-Line^®^ Brown alternative management guidelines (Hy-Line, 2016) but the LED lighting was maintained at 100 lux as the pullets were destined for outdoor access (no natural light was present during rearing). Mechanical ventilation and heating systems were used as needed. Chicks were beak-trimmed using infra-red at the hatchery with a vaccination schedule throughout rearing as per regulatory requirements and standard recommendations including vaccination against Newcastle disease, Marek’s disease, fowl pox, fowl cholera, egg drop syndrome, *Mycoplasma gallisepticum*, *Mycoplasma synoviae*, infectious bronchitis, infectious laryngotracheitis, and avian encephalomyelitis.

### Pullet Sampling

At 15 weeks of age, 45 pullets (*n* = 15 per treatment group) were selected from a random sample of 90 pullets [dissected in Campbell et al. (2020c)] based on balanced live weights across pen replicates. The live weights between the selected pullets varied from 1,250 to 1,370 g (mean ± SE = 1312 ± 4.68 g). The selected hens were killed with CO_2_ and then opened when the movements of the birds had completely ceased. Across a single sampling day, the contents from both ceca were collected, mixed well, transferred to Eppendorf tubes and placed in liquid nitrogen until storage at −80°C at the conclusion of the sampling day.

### Adult Housing

A total of 1,386 pullets were transferred to the free-range facility at the Laureldale farm of the University of New England, Armidale, NSW, Australia at 16 weeks of age and remixed within pen replicates. The hens were housed within the three rearing treatments across 9 adjacent pens (4.8 mL × 3.6 mW) located in a single shed with an indoor density of approximately 9 hens/m^2^ (154 hens/pen). Shade cloth on wire pen dividers was used to visually separate the hens from other pens. The indoor pens contained nest boxes (2 small and 1 large tiered nest box), perches, round hanging feeders and water nipples to fulfill the requirements of the Australian Model Code of Practice for the Welfare of Animals: Domestic Poultry (Primary Industries Standing Committee, 2002). Rice hulls were used as floor litter material with one complete litter replacement mid-way through the flock cycle. The LED lighting gradually increased to 16 h light and 8 h dark by 30 weeks of age with an average pen intensity of 10.0 (0.84 SE) lux (Lutron Light Meter, LX-112850; Lutron Electronic Enterprise CO., Ltd., Taipei, Taiwan) as measured at birds’ eye height from 3 pen locations (front, middle, back) when the pop-holes were closed. The shed was mechanically ventilated with no automated cooling system.

Each of the 9 pens was connected to an outdoor range area (31 mL × 3.6 mW for each pen with a density of approximately 1.4 hens/m^2^). The range was accessed via two pop-hole openings (18 cm W × 36 cm H) per pen. The range area immediately after the pop-holes was 1.1 m length of concrete path, then 1.6 m length of river rock followed by a grassed area with no additional trees or shelter. Each range was visually divided by shade cloth hung along the wire fences. Hens were provided access to the outdoor area from 25 weeks of age (May 2018) for most of the daytime via automatic opening and closing of the pop-holes. The pop-holes opened at 9:15 am and closed after sunset daily (approximately 9 h of ranging time across winter and 11 h ranging after daylight saving time started in October 2018).

### Radio-Frequency Identification of Ranging

All the hens were banded with microchips (Trovan^®^ Unique ID 100 (FDX-A): operating frequency 128 kHz, Microchips Australia Pty Ltd., Keysborough, VIC, Australia) glued into adjustable leg bands (Roxan Developments Ltd., Selkirk, Scotland) to track their movement in and out of the range pop-holes until 64 weeks via radio-frequency identification (RFID) systems. The RFID systems were designed and supported by Microchips Australia Pty Ltd. with equipment developed and manufactured by Dorset Identification B.V. (Aalten, the Netherlands) using Trovan^®^ technology (RFID Systems Ltd., North Ferriby, United Kingdom). The date and time of each tagged bird passing through and in which direction (onto the range, or into the pen) were recorded with a precision of 0.024 s (maximum detection velocity 9.3 m/s). A custom-designed software program written in the “Delphi” language (Bryce Little, Agriculture and Food, CSIRO, St Lucia, QLD, Australia) filtered out unpaired or “false” readings from the RFID data from 56 until 64 weeks of age (54 days of data). The same program summarized the daily data to provide the mean number of hours outside per hen across the sampling period.

### Hen Selection and Sample Collection

At 65 weeks of age, *n* = 91 hens were selected across all pens from all rearing treatments with specific ranging patterns as identified from the RFID data. The hens were categorized as “high outdoor” which were hens that accessed the outdoor range on all the selected days for 5 h 12 min to 9 h on average daily, and “indoor” which were hens that accessed the range on one or zero of the 54 days. Of the selected 91 hens, 44 were indoor hens (control *n* = 14, novelty *n* = 14, structural, *n* = 16), and 47 were high outdoor hens (control *n* = 15, novelty *n* = 17, structural, *n* = 15) totaling *n* = 29 control, *n* = 31 novelty, and *n* = 31 structural hens. Hen selection was balanced across all treatments as best as possible, but some pens within treatments had higher numbers of hens with extreme ranging patterns. The live weight of the selected hens ranged from 1,800 to 2,110 g (mean ± SE = 1966.81 ± 7.39 g). The body weight was not completely balanced as the hens were primarily selected based on their ranging patterns and different rearing enrichment groups; closer body weight between them was prioritized where possible during selection. The same hens were also reported on in Bari et al. (2020c) as they, along with a further 216 hens had post-mortem assessments conducted including internal organ weights, presence of disease/infections, and carcass composition.

At 65 weeks, the selected hens were transported in carrier crates from the free-range facility to a post-mortem facility at the University of New England (∼ 5.5 km distance) just before (∼2 h) the post-mortem. The hens were killed with CO_2_. Immediately after the cessation of all movements, the hens were opened, and the cecal contents were collected and mixed together from both ceca with approximately 200 mg of contents transferred to an Eppendorf tube (2 ml size). These samples were placed on ice immediately after sampling, then transferred to a −20°C freezer half-way through the day. At the end of the sampling day all samples were stored in a −80°C freezer until laboratory analysis.

### DNA Extraction

The DNA of pooled cecal content of both ceca collected at 65 weeks of age was extracted using DNeasy 96 PowerSoil Pro QIAcube HT Kit, (Qiagen, Inc., Doncaster, VIC, Australia) with slight modification. Approximately 65 mg of frozen cecal contents were weighed in a 2 ml Eppendorf tube containing 300 mg of glass beads. Then, 800 μl of solution CD1 was added and vortexed for 5 s. The tubes were then placed in the TissueLyser II for 5 min at a frequency of 30 Hz to disrupt bacterial cells. The tubes were spun briefly and placed in the heat block at 90°C for 10 min. The tubes were vortexed for 5 s followed by centrifuging at 15,000 × *g* for 1 min. Then, the supernatants (approximately 500–650 μl) were transferred to a new 2 ml tube. Later, 250 μl of CD2 were added and the tubes were inverted 3 times followed by centrifuging again at 4,500 × *g* for 5 min. The supernatants were transferred to a new S-Block. The S-Block was placed in the correct position in QIAcube HT. The reactions were loaded into the dedicated cassette with written names. Then the extraction was performed using the QIAcube HT following the manufacturer’s instruction. The quality and quantity of extracted DNA was determined using a NanoDrop spectrophotometer (Nanodrop 8000; Thermo Scientific, Wilmington, DE, United States). The ratios A260/A280 being > 1.8 were considered as of high quality and the extracted DNA were kept at −20°C until required.

### 16S rRNA Gene Sequencing

The V3–V4 region of 16S rRNA genes were amplified using forward primer 16S_341f (TCGTCGGCA GCGTCAGATGTGTATAAGAGACAGCCTACGGGNGGCWG CAG) and reverse primer 16S-805r (GTCTCGTGGGCTC GGAGATGTGTATAAGAGACAGGACTACHVGGGTATCTAA TCC) (Klindworth et al., 2013). The sequencing was performed on an Illumina MiSeq system (2 × 300 bp) at the Ramaciotti Centre for Genomics (UNSW, Sydney, NSW, Australia).

### Short-Chain Fatty Acids Analysis

The cecal short-chain fatty acids (SCFAs) were analyzed according to the method described by Bach Knudsen et al. (1991) and Richardson et al. (1989) with slight modifications. Briefly, frozen cecal samples were defrosted and homogenized keeping them at 4°C overnight. Approximately 0.8 g of the homogenized cecal sample was weighed into a centrifuge tube and 1 mL of internal standard solution (0.01M ethylbutyric acid) was added. The sample with solution was vortexed for 1 min and centrifuged at 15,000 rpm for 20 min at 5°C, with approximately 1 mL supernatant transferred into an 8 mL vial. Using the same method, a blank and an internal standard solution (0.1 mL of 0.1M ethyl butyric acid) were also prepared into 8 mL vials by replacing 1 mL of the supernatant with the same amount of water and standard acid mixture, respectively. Then, 2.5 mL of diethylether and 0.5 mL of concentrated HCl (36%) were added to 8 mL vials containing the supernatant, blank and standard solution, and thoroughly mixed by using a vortex mixer. The mixture was vortexed, and centrifuged at 3,000 rpm at 5°C for 15 min. An aliquot of 400 μL of the supernatant was transferred to a gas chromatograph vial (2 mL) and mixed with 40 μL of *N*-tert-butyldimethlsilyl-*N*-methyltrifuoroacetamide (MTBSTFA) and incubated at 80°C for 20 min. The GC vials were tightened appropriately and left at room temperature for at least 48 h. The cecal SCFAs were measured using a Varian CP3400 CX gas Chromatograph (Varian Analytical Instruments, Palo Alto, CA, United States) after adding approximately 0.5 mL ether into each GC vial. The concentrations of cecal SCFAs were expressed as μmol/g cecal samples.

### Data and Statistical Analyses

A total of 45 cecal content samples of pullets at 15 weeks of age from different rearing enrichments including control, novelty and structural groups were analyzed. A total of 91 cecal content samples of free-range hens at 65 weeks of age from different rearing enrichments including control, novelty and structural, and different ranging patterns (indoor, high outdoor) were analyzed.

The sequence reads’ quality was checked using fastQC v0.11.9 (Babraham Institute, Cambridge, United Kingdom) (Andrews et al., 2014). Quantitative Insights Into Microbial Ecology (QIIME2) (Bolyen et al., 2019) was used for upstream analysis of the sequence employing DADA2 plugin (Callahan et al., 2016) for error correction, filtering, merging pair ends and removing chimeras. The taxonomy was assigned against the Greengenes database (v13_8) using a Naïve Bayes classifier trained against the primers used in this study. The amplicon sequence variants (ASV) table obtained from QIIME2 was then subjected to downstream analysis and visualization with Calypso (Zakrzewski et al., 2017).

The ASV data were normalized by total sum normalization of square-root transformed data in Calypso and the rare taxa were not excluded. The ordination of the groups was done with redundancy analysis (RDA) while a non-parametric multivariate test ANOSIM (Bray-Curtis) was used to test the differences between the groups. Shannon Index, Richness, Chao1, and Simpson’s Index were calculated to analyze alpha diversity among the groups. Linear discriminant analysis Effect Size (LEfSe) was used to identify the differentially abundant representative taxa of each group while differences in individual taxon among different groups was also tested with ANOVA followed by *post hoc* comparisons where significant differences were present. All p-values were adjusted for multiple testing, which is a feature of Calypso (Zakrzewski et al., 2017).

For SCFA analysis, a total of 90 samples were used, of which 30 were from pullets (control *n* = 10, novelty *n* = 10, and structural *n* = 10), and 60 were from adult hens [control hens: *n* = 20 (9 indoor, 11 outdoor); novelty hens: *n* = 20 (8 indoor, 12 outdoor); structural hens: *n* = 20 (12 indoor, 8 outdoor)]. A general linear mixed model (GLMM) was applied to the pullet cecal SCFA data with rearing enrichments as a fixed effect and Bird ID nested within Pen nested within rearing enrichments as a random effect. For the adult hens, cecal SCFA data, a GLMM was applied with rearing enrichments and ranging patterns as fixed effects and Bird ID nested within Pen and Pen nested within rearing enrichments and ranging patterns as random effects. The interaction term for the adult data was not included to better match the microbiota analyses from these birds. Data were Log_10_ transformed for formic acid and lactic acid content in the pullet SCFAs. For the adult hen SCFAs, the formic acid values were Log_10_ transformed and the lactic acid values were square-root transformed. Where significant differences were present, *post hoc* Student’s t-tests were applied to the least squares means. All these statistical analyses were conducted in JMP 14.0^®^ (SAS Institute, Cary, NC, United States) with α set at 0.05.

## Results

### Cecal Microbiota of Pullets

**FIGURE 1:**
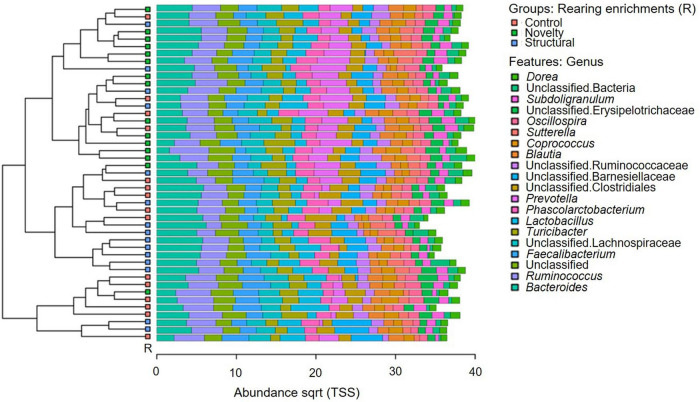
Microbial community composition of pullets at 15 weeks of age from different rearing enrichments (control, novelty, structural). The top 20 abundant microbial genera are shown (clustered bar chart) in different enrichment groups of pullets.

The microbial community composition of the pullets is shown in Figure 1. The predominant microbiota genera included *Dorea*, unclassified bacteria, *Subdoligranulum*, unclassified Erysipelotrichaceae, *Oscillospira, Sutterella, Coprococcus, Blautia*, unclassified Ruminococcaceae, unclassified Barnesiellaceae, unclassified Clostridiales, *Prevotella, Phascolarctobacterium, Lactobacillus, Turicibacter*, unclassified Lachnospiraceae *Faecalibacterium*, Unclassified, *Ruminococcus*, and *Bacteroides.*

**FIGURE 2:**
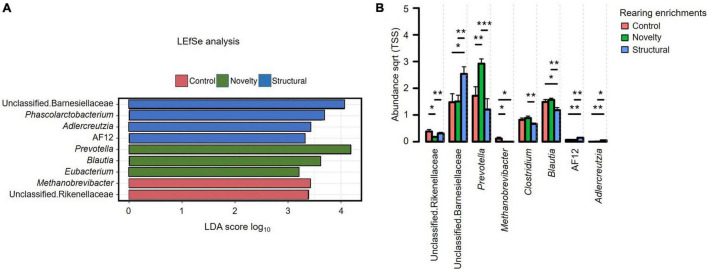
**(A)** Relative abundance of cecal microbiota of pullets at 15 weeks of age from different rearing enrichments (control, novelty, structural) showing linear discriminant analysis (LDA) effect size method (LEfSe). **(B)** Differences in the relative abundance of cecal microbiota of pullets at 15 weeks of age from different rearing enrichments (control, novelty, structural) at genus level. One-way ANOVA followed by *post hoc* comparisons between rearing enrichment groups showed differences *P ≤ 0.05, **P ≤ 0.01, ***P ≤ 0.001.

The sequence reads per sample revealed no significant differences between the treatment groups (*P* = 0.35) with a minimum read of 19,840 and a maximum read of 64,330 sequences. The Linear Discriminant (LDA) Effect Size (LEfSe) analysis (Figure 2A) showed the differentially abundant explanatory microbiota genera in the structural group included unclassified Barnesiellaceae, *Phascolarctobacterium, Adlercreutzia*, and AF12 with *Prevotella, Blautia* and *Eubacterium* predominant in the novelty group, and *Methanobrevibacter* and unclassified Rikenellaceae explanatory in the control group. Explanatory taxa (genera in our case) explain the differences between the groups. Figure 2B displays the relative abundance of cecal microbiota of pullets at 15 weeks of age from different rearing enrichments at the genera level with all bacterial groups showing a significant effect of rearing enrichment treatment (all *P* < 0.05). The *Methanobrevibacter* genus was more abundant in the control group than the other two groups (*P* ≤ 0.05), and *Prevotella* was more abundant in the novelty group than the control (*P* ≤ 0.01) and structural groups (*P* ≤ 0.001) of pullets. The *Clostridium* genus was more abundant in the novelty group than the structural pullets only (*P* ≤ 0.01). The *Blautia* was less abundant in the structural group than in both novelty and control pullets (*P* ≤ 0.01), but there was no difference in the *Blautia* abundance between the control and novelty pullets. The *Adlercreutzia* genus was more abundant in the structural group of pullets than the control (*P* ≤ 0.01) and novelty (*P* ≤ 0.05) groups of pullets. The unclassified Rikenellaceae was more abundant in the control group than the novelty pullets (*P* ≤ 0.05), and more abundant in the structural group than the novelty pullets (*P* ≤ 0.01). The unclassified Barnesiellaceae was more abundant in the structural group than the novelty (*P* ≤ 0.05) and control (*P* ≤ 0.01) pullets. The genus AF12 was more abundant in the structural group than the novelty (*P* ≤ 0.01) and control groups (*P* ≤ 0.01) of pullets.

**FIGURE 3:**
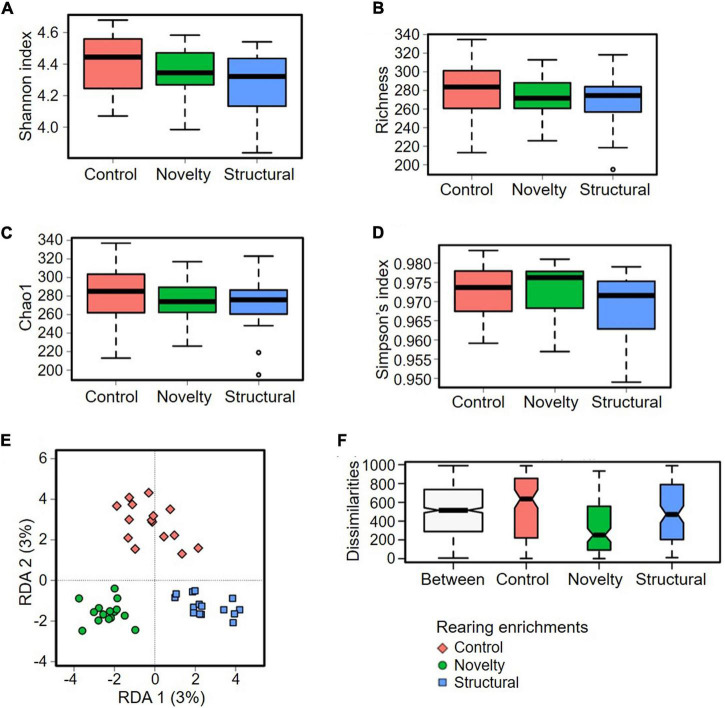
Microbial diversity of cecal microbiota of pullets at 15 weeks of age from different rearing enrichments (control, novelty, structural) at ASV level. Alpha diversity: **(A)** Shannon index, *P* = 0.17; **(B)** Richness, *P* = 0.51; **(C)** Chao1, *P* = 0.54; **(D)** Simpson’s index, *P* = 0.34; and Beta diversity: **(E)** Redundancy analysis (RDA), *P* = 0.002; **(F)** ANOSIM (Bray-Curtis), *P* = 0.004.

Two multivariate analyses of beta diversity at ASV level, redundancy analysis (RDA) (Figure 3E) (*P* = 0.002) and ANOSIM (Bray–Curtis) (R = 0.112, *P* = 0.004) showed significant differences between the groups of rearing enrichment treatments (Figures 3E,F). However, there was no difference in the alpha diversity indices at ASV level among the rearing enrichment groups as assessed by Shannon index (*P* = 0.17), Richness (*P* = 0.51), Chao1 (*P* = 0.54) and Simpson’s index (*P* = 0.34) (Figures 3A–D).

### Cecal Microbiota of Free-Range Hens

**FIGURE 4:**
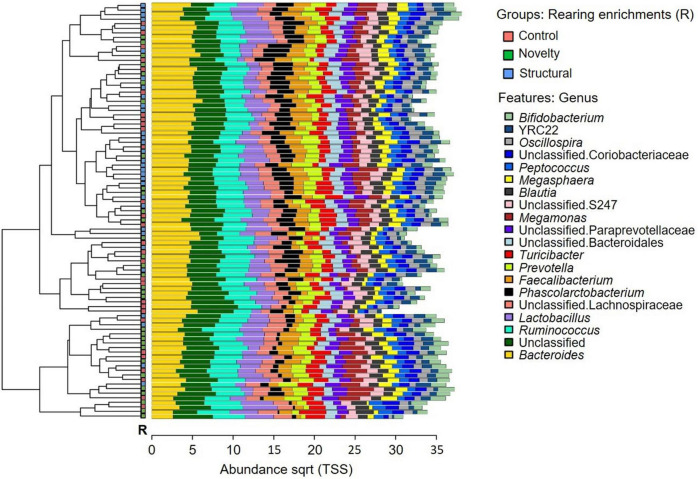
Microbial community composition of free-range hens at 65 weeks of age from different rearing enrichments (control, novelty, structural). Clustered bar chart showing 20 most abundant genera.

**FIGURE 5:**
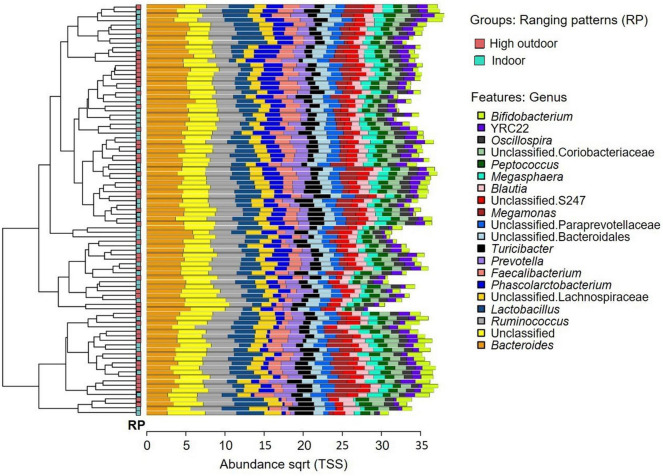
Microbial community composition of free-range hens at 65 weeks of age from different ranging patterns (high outdoor, indoor). Clustered bar chart showing 20 most abundant genera.

The microbial community composition of the free-range hens from different rearing enrichments is shown in Figure 4; and from different ranging patterns in Figure 5. The predominant microbiota genera from the rearing enrichment groups and ranging patterns included *Bifidobacterium*, YRC22, *Oscillospira*, unclassified Coriobacteriaceae, *Peptococcus, Megasphaaera, Blautia*, unclassified S247, *Megamonas*, unclassified Paraprevotellaceae, unclassified Bacteroidales, *Turicibacter, Prevotella, Faecalibacterium, Phascolarctobacterium*, unclassified Lachnospiraceae, *Lactobacillus, Ruminococcus*, Unclassified, and *Bacteroides.*

The sequence reads per sample revealed no significant differences between the rearing enrichment groups (*P* = 0.20) with a minimum read of 14,055 and a maximum read of 52,056 sequences. The sequence reads per sample was also not significant between the ranging groups (*P* = 0.91) with a minimum read of 14,055 and a maximum read of 52,056 sequences.

**FIGURE 6:**
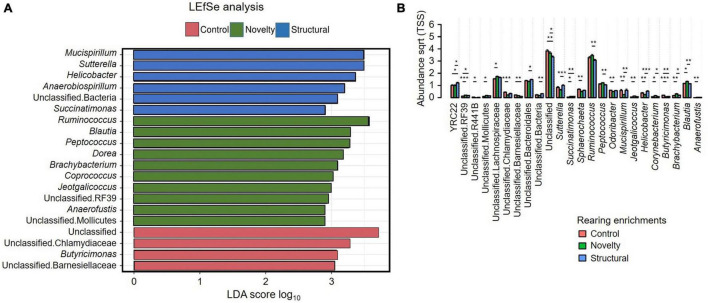
**(A)** Relative abundance of cecal microbiota of free-range hens at 65 weeks of age from different rearing enrichments (control, novelty, structural) showing linear discriminant analysis (LDA) effect size method (LEfSe). **(B)** Relative abundance of cecal microbiota of free-range hens at 65 weeks of age from different rearing enrichments (control, novelty, structural) at genus level. One-way ANOVA followed by *post hoc* comparisons between rearing enrichment groups showed differences *P ≤ 0.05, **P ≤ 0.01, ***P ≤ 0.001.

Figure 6A shows the LEfSe analysis results that indicated the differentially abundant explanatory microbiota genera from the rearing enrichment groups. LEfSe analysis showed *Mucispirillum, Sutterella, Helicobacter, Anaerobiospirillum*, unclassified Bacteria, and *Succinatimonas* were the characteristic genera from the structural group, *Ruminococcus, Blautia, Peptococcus, Dorea, Brachybacterium, Coprococcus, Jeotgalicoccus*, unclassified RF39, *Anaerofustis*, and unclassified Mollicutes from the novelty group, and Unclassified, unclassified Chlamydiaceae, *Butyricimonas*, and unclassified Barnesiellaceae from the control group of rearing treatments. Figure 6B shows the relative abundance of cecal microbiota of free-range hens at 65 weeks of age from different rearing enrichment treatments at the genera level where there were significant effects of rearing enrichment treatments across all bacterial groups (all *P* < 0.05). The *Sutterella* genus was more abundant in the structural hens than in the novelty hens (*P* ≤ 0.001), but the control group did not differ. The *Succinatimonas* was more predominant in the structural (*P* ≤ 0.01) and novelty groups (*P* ≤ 0.05) than the control hens. The *Sphaerochaeta* genus was more abundant in the control group than the novelty hens (*P* ≤ 0.05), but there was no difference between the novelty and structural hens. *Ruminococcus* was more abundant in the novelty hens (*P* ≤ 0.01) than the structural, but did not differ between control and structural hens. The *Peptococcus* genus was also more abundant in the novelty hens than the structural group (*P* ≤ 0.01). The *Mucispirillum* was more abundant in both the control and structural hens than the novelty group (*P* ≤ 0.01). The *Odoribacter* genus was more abundant in the control group than the novelty (*P* ≤ 0.01). The *Jeotgalicoccus* was more abundant in the novelty hens than the control hens (*P* ≤ 0.01), but did not differ between the novelty and structural hens. The *Corynebacterium* was more abundant in the novelty hens than both the control and structural hens (*P* ≤ 0.05). *Butyricimonas* was more abundant in the control hens than the novelty (*P* ≤ 0.001) and structural hens (*P* ≤ 0.01). The *Brachybacterium* and *Blautia* genera were more predominant in the novelty hens than the control (*P* ≤ 0.01) and structural hens (*P* ≤ 0.05), and the *Anaerofustis* was more predominant in the novelty hens than the control hens (*P* ≤ 0.01) but did not differ between the novelty and structural hens (Figure 6B). The unclassified RF39 had significantly lower abundance in the control hens than in both the novelty and structural groups (*P* ≤ 0.05), but there was no difference in the unclassified RF39 abundance between the novelty and structural hens. The control group had more abundant unclassified R441B bacteria than the control (*P* ≤ 0.05) hens. Overall, the significantly more abundant bacteria were YRC22 in structural, *Blautia, Brachybacterium* and *Corynebacterium* in novelty and *Butyricimonas* in control hens; and the significantly less abundant bacteria were Unclassified in structural, *Succinatimonas* in control, and *Mucispirillum* and *Helicobacter* in novelty.

**FIGURE 7:**
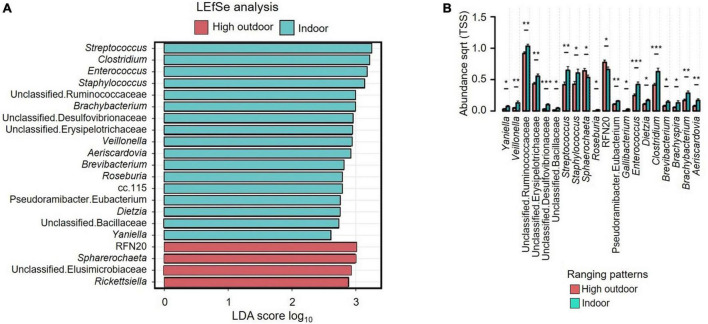
**(A)** Relative abundance of cecal microbiota of free-range hens at 65 weeks of age from different ranging patterns (high outdoor, indoor) showing linear discriminant analysis (LDA) effect size method (LEfSe). The most abundant genera of microbiota from different ranging groups are shown. **(B)** Relative abundance of cecal microbiota of free-range hens at 65 weeks of age from different ranging patterns (high outdoor, indoor) at genus level. One-way ANOVA followed by *post hoc* comparisons between ranging patterns showed differences *P ≤ 0.05, **P ≤ 0.01, ***P ≤ 0.001.

Figure 7A shows the LEfSe analysis results that indicated the explanatory microbiota genera in the hens from different ranging patterns. The indoor hens had *Streptococcus*, *Clostridium, Enterococcus, Staphylococcus*, unclassified Ruminococcaceae, *Brachybacterium*, unclassified Desulfovibrionaceae, unclassified Erysipelotrichaceae, *Veillonella, Aeirscardovia, Brevibacterium, Roseburia*, cc.115, Pseudoramibacter Eubacterium, *Dietzia*, unclassified Bacillaceae and *Yaniella* as the explanatory genera whereas in the high outdoor group RFN20, *Sphaerochaeta*, unclassified Elusimicrobiaceae, and *Rickettsiella* were the explanatory bacteria. Figure 7B shows that the indoor hens had *Yaniella* (*P* ≤ 0.05), *Veillonella (P* ≤ 0.01), *Streptococcus (P* ≤ 0.01), *Staphylococcus (P* ≤ 0.05), *Roseburia (P* ≤ 0.05), *Gallibacterium (P* ≤ 0.05), *Enterococcus (P* ≤ 0.001), *Dietzia (P* ≤ 0.05), *Clostridium (P* ≤ 0.001), *Brevibacterium (P* ≤ 0.05), *Brachyspira (P* ≤ 0.05), *Brachybacterium (P* ≤ 0.01), and *Aeriscardovia (P* ≤ 0.05) genera in greater abundance than the high outdoor hens, but the *Sphaerochaeta* were more predominant in the high outdoor hens than the indoor hens (*P* ≤ 0.05). Also, all of the unclassified genera including unclassified Ruminococcaceae (*P* ≤ 0.01), unclassified Erysipelotrichaceae (*P* ≤ 0.01), unclassified Desulfovibrionaceae (*P* ≤ 0.001), unclassified Bacillaceae (*P* ≤ 0.05) and Pseudoramibacter Eubacterium (*P* ≤ 0.01) were more predominant in the indoor hens than the high outdoor hens. However, the RFN20 bacteria was more predominant (*P* ≤ 0.05) in the high outdoor hens than the indoor hens.

**FIGURE 8:**
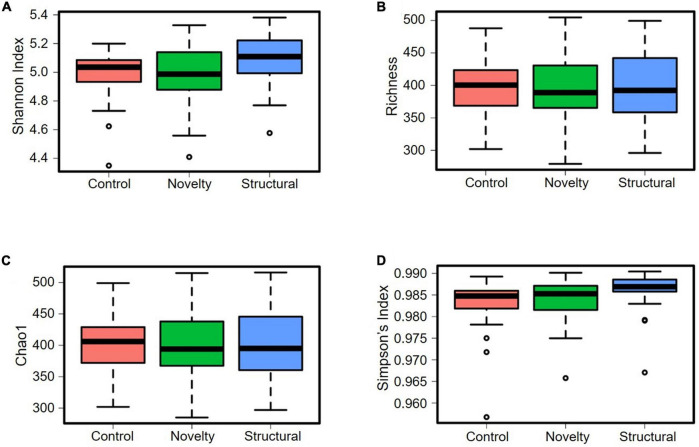
Microbial diversity (alpha-diversity) of cecal microbiota of free-range hens at 65 weeks of age from different rearing enrichments (control, novelty, structural) at ASV level. **(A)** Shannon index, *P* = 0.07; **(B)** Richness, *P* = 0.74; **(C)** Chao1, *P* = 0.72; **(D)** Simpson’s index, *P* = 0.07.

**FIGURE 9:**
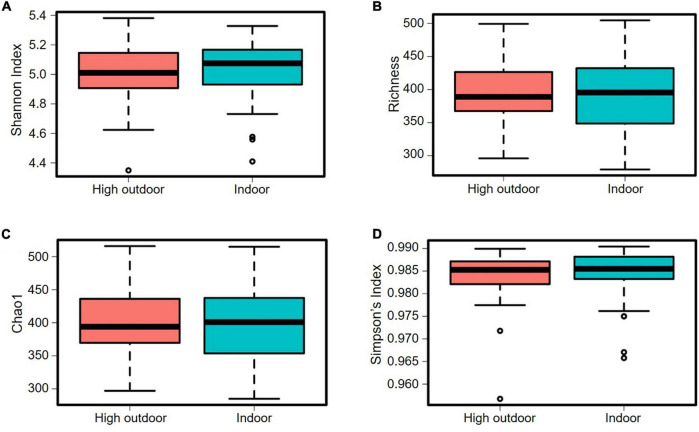
Microbial diversity (alpha-diversity) of cecal microbiota of free-range hens at 65 weeks of age from different ranging patterns (high outdoor, indoor) at ASV level. **(A)** Shannon index, *P* = 0.74; **(B)** Richness, *P* = 0.66; **(C)** Chao1, *P* = 0.66; **(D)** Simpson’s index, *P* = 0.54.

**FIGURE 10:**
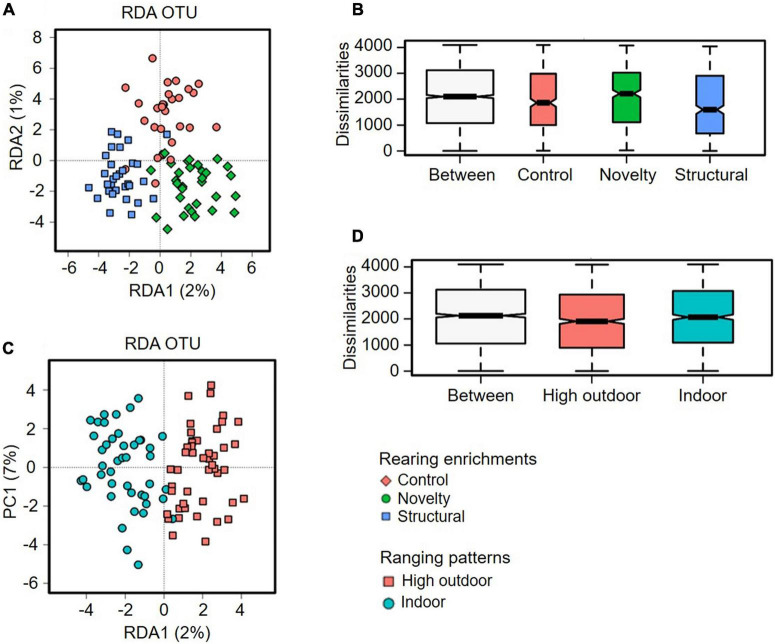
Microbial diversity (Beta-diversity) of cecal microbiota of free-range hens at 65 weeks of age from different rearing enrichments (control, novelty, structural) and ranging patterns (high outdoor, indoor) at ASV level. **(A)** Redundancy analysis (RDA), *P* = 0.001; **(B)** ANOSIM (Bray-Curtis), *P* = 0.001. Microbial diversity of cecal microbiota of free-range hens at 65 weeks of age from different ranging patterns (high outdoor, indoor); **(C)** Redundancy analysis (RDA), *P* = 0.006; **(D)** ANOSIM (Bray–Curtis), *P* = 0.008.

The cecal microbiota diversity of free-range hens at ASV level are shown in Figures 8–10. The alpha diversity at ASV level indicated no significant difference in microbiota populations within each of the rearing enrichment groups as assessed by Shannon index (*P* = 0.06), Richness (*P* = 0.74), Chao1 (*P* = 0.72) and Simpson’s index (*P* = 0.07) (Figures 8A–D). The alpha diversity indicated no significant variation in the bacterial populations within each of the two range use groups of hens (Figures 9A–D) as assessed by Shannon index (*P* = 0.74), Richness (*P* = 0.66), Chao1 (*P* = 0.66) and Simpson’s index (*P* = 0.54). The differences in beta diversity for rearing enrichments (Figures 10A,B) indicated by Redundancy analysis (RDA) (*P* = 0.001) and ANOSIM (Bray–Curtis) (R = 0.06, *P* = 0.001) showed significant variation in the bacteria between the rearing enrichment treatments. Similarly, the differences in beta diversity for ranging patterns (Figures 10C,D) indicated by RDA (*P* = 0.006) and ANOSIM (Bray–Curtis) (R = 0.04, *P* = 0.008) showed significant variation in the bacterial diversity between the indoor and high outdoor hens.

### Short-Chain Fatty Acids

For the pullet SCFAs, the propionic acid content was higher in the novelty pullets than the control pullets (*F*_2,27_ = 4.04,*P* = 0.03) (Table 1). For the other SCFAs including formic acid, acetic acid, isobutyric acid, butyric acid, isovaleric acid, valeric acid, lactic acid, and succinic acid content, rearing enrichments did not have a significant effect (all *P*≥0.10) (Table 1).

**TABLE 1:** Least squares means (LSM) ± SEM of short-chain fatty acids (SCFAs) (μmol/g) in cecal contents of pullets and adult free-range hens from different rearing enrichment treatments (control, novelty, structural) and ranging patterns (indoor, high outdoor).

Parameters	Rearing enrichments			F ratio, *P* value	Ranging patterns		F ratio, *P* value
	Control	Novelty	Structural		Indoor	High Outdoor	
**Pullets**							
Formic acid	0.68 ± 0.10	0.80 ± 0.10	0.72 ± 0.10	F_2, 27_ = 0.09, 0.92	−	−	−
Acetic acid	61.60 ± 5.95	78.75 ± 5.95	63.70 ± 5.95	F_2, 27_ = 2.48, 0.10	−	−	−
Propionic acid	9.89 ± 0.86^b^	13.19 ± 0.86^a^	10.72 ± 0.8^ab^	F_2, 27_ = 4.04, 0.03	−	−	−
Isobutyric acid	0.55 ± 0.06	0.47 ± 0.06	0.53 ± 0.06	F_2, 27_ = 0.54, 0.59	−	−	−
Butyric acid	14.12 ± 1.82	16.94 ± 1.82	4.24 ± 1.82	F_2, 27_ = 0.77, 0.47	−	−	−
Isovaleric acid	0.17 ± 0.02	0.14 ± 0.02	0.18 ± 0.02	F_2, 27_ = 0.78, 0.47	−	−	−
Valeric acid	1.34 ± 0.13	1.49 ± 0.13	1.33 ± 0.13	F_2, 27_ = 0.44, 0.65	−	−	−
Lactic acid	1.08 ± 0.97	2.87 ± 0.97	1.15 ± 0.97	F_2, 27_ = 0.35, 0.71	−	−	−
Succinic acid	1.42 ± 0.16	0.97 ± 0.16	1.35 ± 0.16	F_2, 27_ = 2.24, 0.13	−	−	−
**Adult hens**							
Formic acid	1.01 ± 0.08	0.79 ± 0.08	0.80 ± 0.08	F_2_,_18_ = 1.76, 0.20	0.88 ± 0.07	0.84 ± 0.07	F_1_,_19_ = 0.02, 0.89
Acetic acid	66.75 ± 5.42	82.22 ± 5.47	77.06 ± 5.49	F_2_,_13_ = 2.10, 0.16	73.50 ± 4.47	77.19 ± 4.45	F_1_,_13_ = 0.34, 0.57
Propionic acid	27.37 ± 2.32	32.53 ± 2.34	31.92 ± 2.35	F_2_,_13_ = 1.47, 0.27	29.28 ± 1.91	31.93 ± 1.91	F_1_,_14_ = 0.95, 0.35
Isobutyric acid	1.28 ± 0.18	0.98 ± 0.18	1.03 ± 0.18	F_2_,_14_ = 0.80, 0.47	1.09 ± 0.15	1.10 ± 0.15	F_1_,_14_ = 0.003, 0.96
Butyric acid	11.23 ± 1.12	14.51 ± 1.13	13.55 ± 1.13	F_2_,_15_ = 2.26, 0.14	12.16 ± 0.93	14.03 ± 0.91	F_1_,_16_ = 2.05, 0.17
Isovaleric acid	0.45 ± 0.09	0.30 ± 0.09	0.32 ± 0.10	F_2_,_14_ = 0.68, 0.52	0.36 ± 0.08	0.36 ± 0.08	F_1_,_14_ = 0.0002, 0.99
Valeric acid	2.15 ± 0.24	2.47 ± 0.24	2.10 ± 0.24	F_2_,_17_ = 0.72, 0.50	2.19 ± 0.20	2.28 ± 0.19	F_1_,_17_ = 0.11, 0.74
Lactic acid	1.62 ± 1.05	1.47 ± 1.06	2.15 ± 1.05	F_2_,_17_ = 0.34, 0.72	1.47 ± 0.87	2.02 ± 0.85	F_1_,_18_ = 0.44, 0.52
Succinic acid	1.04 ± 0.15	0.99 ± 0.15	1.40 ± 0.15	F_2_,_17_ = 2.03, 0.16	1.17 ± 0.13	1.12 ± 0.13	F_1_,_17_ = 0.10, 0.76

*^a,b^Dissimilar superscript letters indicate significant differences between rearing enrichment treatments (P < 0.05).*

There were no significant effects of both rearing enrichments (all *P*≥0.14) and ranging patterns (all *P*≥0.17) for the SCFAs including formic acid, acetic acid, propionic acid, isobutyric acid, butyric acid, isovaleric acid, valeric acid, lactic acid, and succinic acid content in the ceca of free-range hens.

## Discussion

This study was conducted to assess whether different types of environmental enrichments provided during the rearing period of laying hen pullets would affect their cecal microbiota composition and whether these rearing enrichments would continue to have impact on microbiota in adult free-range hens. Additionally, this study assessed the relationship between cecal microbiota composition and individual variation in range use patterns. Overall, the cecal microbiota abundance varied between the enriched and non-enriched pullets with rearing differences persisting in the adult hens. Cecal microbiota abundance also varied between hens with different ranging patterns. Assessment of short-chain fatty acids detected minimal differences based on rearing treatments in pullets and no difference in adults, or any association with adult ranging patterns. Environmental variation has impact on laying hen microbiota, but the functional impacts of this variation remain to be determined.

The microbiota variation in the pullets might have been related to bacterial exposure from different novel objects that were placed in the shed and changed at weekly intervals. The addition of perching structures may have also had impact through accumulation of feces on the solid surfaces. Thus, the effects could have been solely related to external environmental variation in bacterial exposure. Alternatively, or in combination with external bacterial variation, there may have been an interplay between the behavior of the pullets and the colonization process for their microbial communities. Recent evidence from pigs, reported that enriched housing affected microbiota colonization relative to standard control housing (Wen et al., 2021), although this effect was not confirmed across other research (Saladrigas-García et al., 2021). The inclusion of enrichment in the pens resulted in some variation across the rearing period in exhibited behaviors (Campbell et al., 2022), responses to fear tests (Campbell et al., 2021), and body weight (Campbell et al., 2020c). In the week following the sample collection when the pullets were transferred to the laying facility, there were also rearing treatment differences in perching behavior in the new pens (Campbell et al., 2020c). In contrast, there were other behavioral test measures and post-mortem assessment of internal organs that did not show any rearing enrichment effects (Campbell et al., 2020c). Other research with laying hens has shown gut microbiota composition varies relative to different genetic lines selected for high or low feather pecking behavior (Birkl et al., 2018; van der Eijk et al., 2019). When microbiota is transplanted between the lines, responses in subsequent behavioral testing are affected demonstrating the interaction that is present between gut microbiota and behavioral phenotype (van der Eijk et al., 2020). Similar evidence has been reported in quail selected for high or low emotional reactivity where transplantation between the lines can impact reactivity in subsequent emotional testing, modulating expected responses for the selected line (Kraimi et al., 2019a). Research in quail has also shown probiotics can affect responses in cognitive testing (Parois et al., 2017). Although there were microbiota differences among the rearing treatment groups, and other behavioral differences among the pullets, the causal link between the two is still uncertain with minimal effects of rearing enrichments detected in the short-chain fatty acids. Thus, there is great scope to conduct further studies to identify the causes and consequences of microbiota variation in relation to environmental enrichment and potential behavioral and health effects of the microbiota-gut-brain axis in poultry (Villageliu and Lyte, 2017; Kraimi et al., 2019b).

Rearing enrichments also affected the cecal microbiota abundance in adult hens. Upon transfer from the rearing facility, the adult hens were all housed in the same shed with fresh litter, although they were separated into adjacent pens. Previous research has shown microbiota composition will change with age (Videnska et al., 2014), particularly following transfer from the rearing to the laying facility (Joat et al., 2021), but will be more similar between hens from the same housing shed compared with hens from different sheds of the same type of housing system (Schreuder et al., 2020). The adult hens retained rearing enrichment differences through to the end of the production cycle, although the specific phylogenetic groups that differed changed across the pullet and adult sampling points. In accordance with these microbiota differences, the rearing treatments had long-term impacts on other behavior, welfare, and egg quality measures in the birds (Bari et al., 2020a,b,c, 2021; Campbell et al., 2020b,^2022^), although not on the short-chain fatty acids analyzed in the current study. These enrichment treatment differences support sustained effects of rearing environments regardless of the similar housing conditions experienced as adults. In terms of behavior, there were differences in how the hens used the range with the structural hens ranging for the longest time, the novelty hens showing the fewest range visits, and both the structural and novelty hens spent longer on the range during each individual visit relative to the control hens (Campbell et al., 2020c). These ranging differences likely resulted in corresponding dietary differences across treatment groups in how often they consumed the formulated feed located inside the shed. Hens on the range would have had the opportunity to consume vegetation, insects, and grit with diet demonstrated to directly affect microbial communities (Borrelli et al., 2017; Zhou et al., 2021). The control hens also showed the poorest plumage condition across time indicating higher levels of feather pecking in these birds (Bari et al., 2020b). Similar to the pullets, the casual relationship between rearing enrichments, microbiota and other behavioral and welfare measures is unable to be confirmed from this study but warrants further investigation to understand these associations.

There was distinct variation in abundance of bacterial genera between the indoor and high outdoor hens showing these two groups could be differentiated by their microbiota profiles. While several previous studies have compared housing system effects on microbiota, including birds housed in indoor versus outdoor systems (Cui et al., 2017; Chen et al., 2019; Hubert et al., 2019; Schreuder et al., 2020, 2021; Seidlerova et al., 2020), to the authors’ knowledge, this is the first study that has showed differences in these indoor/outdoor subpopulations of birds from the same communal housing system. These microbiota results align with other behavioral evidence distinguishing hens that range frequently versus remain inside the shed within the same free-range flock. Studies across multiple experimental and commercial systems have shown greater fear and anxiety in the indoor-preferring birds (reviewed in Campbell et al., 2020a). In an associated study conducted on a subsample of birds which included the individuals sampled in the current study, the indoor hens showed poorer plumage condition, more comb wounds, but higher body weight than the high outdoor hens (Bari et al., 2020c). The differences in ranging patterns also likely led to differences in diet as hens outside had the opportunity to forage for insects and consume grit. As per the results with rearing enrichments, the casual relationship between the microbiota profiles and ranging patterns was unable to be established from the current study and is an area for further research to better understand and refine management practices for free-range hens. Similarly, individual resource use patterns by hens within an indoor system, such as the distinct variation demonstrated in aviary-housed hens and the areas of the system they frequent (Campbell et al., 2016; Rufener et al., 2018), may also correspond with variation in gut microbiota, but this would need to be confirmed.

## Conclusion

The different enrichments during rearing influenced the cecal microbiota composition of the pullets and this effect remained in the adult hens when they were moved to the same free-range shed and housed in identical pens. The subsequent individual differences in the ranging patterns also corresponded with differences in microbiota profiles with a greater abundance of different types of cecal microbiota genera in the indoor hens compared with the high outdoor ranging hens. Short-chain fatty acids predominantly did not differ across enrichment and ranging groups and further research is needed to understand the causal relationships among the microbiota differences that were found.

## Data Availability Statement

The data presented in the study are deposited in the NCBI repository, accession number PRJNA786377 (https://www.ncbi.nlm.nih.gov/bioproject/786377).

## Ethics Statement

The animal study was reviewed and approved by the University of New England Animal Ethics Committee (AEC17-092).

## Author Contributions

MB and DC conceived and designed the experiment. CK initiated and assisted in the cecal sample collection. MB collected and analyzed the data, prepared the figures and tables, and drafted the manuscript. SK and YB contributed to the data processing and analysis. S-BW provided expert guidance. DC wrote sections of the manuscript. All authors reviewed the manuscript critically and approved the final version.

## Conflict of Interest

The authors declare that the research was conducted in the absence of any commercial or financial relationships that could be construed as a potential conflict of interest.

## Publisher’s Note

All claims expressed in this article are solely those of the authors and do not necessarily represent those of their affiliated organizations, or those of the publisher, the editors and the reviewers. Any product that may be evaluated in this article, or claim that may be made by its manufacturer, is not guaranteed or endorsed by the publisher.

## References

[B1] Adhikari B. Jun S.-R. Kwon Y. M. Kiess A. S. Adhikari P. (2020). Effects of housing types on cecal microbiota of two different strains of laying hens during the late production phase. *Front. Vet. Sci.* 7:331. 10.3389/fvets.2020.00331 32656252 PMC7324799

[B2] Andrews S. F. Krueger F. Seconds-Pichon A. Biggins F. Wingett S. F. (2014). A quality control tool for high throughput sequence data. *Babraham Bioinformatics* Available online at: https://www.bioinformatics.babraham.ac.uk/projects/fastqc/

[B3] Bach Knudsen K. E. Jensen B. B. Andersen J. O. Hansen I. (1991). Gastrointestinal implications in pigs of wheat and oat fractions: 2. Microbial activity in the gastrointestinal tract. *Br. J. Nutr.* 65 233–248. 10.1079/bjn19910083 1645993

[B4] Bari M. S. Allen S. S. Mesken J. Cohen-Barnhouse A. M. Campbell D. L. M. (2021). Relationship between range use and fearfulness in free-range hens from different rearing enrichments. *Animals* 11:300. 10.3390/ani11020300 33503915 PMC7912001

[B5] Bari M. S. Cohen-Barnhouse A. M. Campbell D. L. M. (2020a). Early rearing enrichments influenced nest use and egg quality in free-range laying hens. *Animal* 4 1249–1257. 10.1017/S1751731119003094 31907088

[B6] Bari M. S. Downing J. A. Dyall T. R. Lee C. Campbell D. L. M. (2020b). Relationships between rearing enrichments, range use, and an environmental stressor for free-range laying hen welfare. *Front. Vet. Sci.* 7:480. 10.3389/fvets.2020.00480 32923465 PMC7457091

[B7] Bari M. S. Laurenson Y. C. S. M. Cohen-Barnhouse A. M. Walkden-Brown S. W. Campbell D. L. M. (2020c). Effects of outdoor ranging on external and internal health parameters for hens from different rearing enrichments. *PeerJ* 8:e8720. 10.7717/peerj.8720 32185113 PMC7061908

[B8] Bavananthasivam J. Astill J. Matsuyama-Kato A. Taha-Abdelaziz K. Shojadoost B. Sharif S. (2021). Gut microbiota is associated with protection against Marek’s disease virus infection in chickens. *Virology* 553 122–130. 10.1016/j.virol.2020.10.011 33271490

[B9] Birkl P. Bharwani A. Kjaer J. B. Kunze W. McBride P. Forsythe P. (2018). Differences in cecal microbiome of selected high and low feather-pecking laying hens. *Poult. Sci.* 97 3009–3014. 10.3382/ps/pey167 29800328 PMC6093748

[B10] Bolyen E. Rideout J. R. Dillon M. R. Bokulich N. A. Abnet C. C. Al-Ghalith G. A. (2019). Reproducible, interactive, scalable and extensible microbiome data science using QIIME 2. *Nat. Biotechnol.* 37 852–857. 10.1038/s41587-019-0209-9 31341288 PMC7015180

[B11] Borda-Molina D. Iffland H. Schmid M. Müller R. Schad S. Seifert J. (2021). Gut microbial composition and predicted functions are not associated with feather pecking and antagonistic behavior in laying hens. *Life* 11:235. 10.3390/life11030235 33809351 PMC8001194

[B12] Borrelli L. Coretti L. Dipineto L. Bovera F. Menna F. Chiariotti L. (2017). Insect-based diet, a promising nutritional source, modulates gut microbiota composition and SCFAs production in laying hens. *Sci. Rep.* 7:16269. 10.1038/s41598-017-16560-6 29176587 PMC5701250

[B13] Bray H. J. Ankeny R. A. (2017). Happy chickens lay tastier eggs: motivations for buying free-range eggs in Australia. *Anthrozoös* 30 213–226. 10.1080/08927936.2017.1310986

[B14] Callahan B. J. McMurdie P. J. Rosen M. J. Han A. W. Johnson A. J. Holmes S. P. (2016). DADA2: High-resolution sample inference from Illumina amplicon data. *Nat. Methods* 13 581–583. 10.1038/nmeth.3869 27214047 PMC4927377

[B15] Campbell D. L. M. Whitten J. M. Slater E. Lee C. (2021). Rearing enrichments differentially modified hen personality traits and reduced prediction of range use. *Anim. Behav.* 179 97–109. 10.1016/j.anbehav.2021.06.024

[B16] Campbell D. L. M. De Haas E. N. Lee C. (2019). A review of environmental enrichment for laying hens during rearing in relation to their behavioral and physiological development. *Poult. Sci.* 98 9–28. 10.3382/ps/pey319 30107615 PMC6347129

[B17] Campbell D. L. M. Bari M. S. Rault J.-L. (2020a). Free-range egg production: its implications for hen welfare. *Anim. Prod. Sci.* 61 848–855. 10.1071/AN19576

[B18] Campbell D. L. M. Belson S. Dyall T. R. Lea J. M. Lee C. (2022). Impacts of rearing enrichments on pullets and free-range hens’ positive behaviors across the flock cycle. *Animals* 12:280. 10.3390/ani12030280 35158604 PMC8833614

[B19] Campbell D. L. M. Dyall T. R. Downing J. A. Cohen-Barnhouse A. M. Lee C. (2020c). Rearing enrichments affected ranging behavior in free-range laying hens. *Front. Vet. Sci.* 7:446. 10.3389/fvets.2020.00446 32923462 PMC7457029

[B20] Campbell D. L. M. Gerber P. F. Downing J. A. Lee C. (2020b). Minimal effects of rearing enrichments on pullet behaviour and welfare. *Animals* 10:314. 10.3390/ani10020314 32085379 PMC7070349

[B21] Campbell D. L. M. Karcher D. M. Siegford J. M. (2016). Location tracking of individual laying hens housed in aviaries with different litter substrates. *Appl. Anim. Behav. Sci.* 184 74–79. 10.1016/j.applanim.2016.09.001

[B22] Chen S. Xiang H. Zhang H. Zhu X. Wang D. Wang J. (2019). Rearing system causes changes of behavior, microbiome, and gene expression of chickens. *Poult. Sci.* 98 3365–3376. 10.3382/ps/pez140 30916350

[B23] Cui Y. Wang Q. Liu S. Sun R. Zhou Y. Li Y. (2017). Age-related variations in intestinal microflora of free-range and caged hens. *Front. Microbiol.* 8:1310. 10.3389/fmicb.2017.01310 28744281 PMC5504432

[B24] Diaz Carrasco J. M. Casanova N. A. Fernández Miyakawa M. E. (2019). Microbiota, gut health and chicken productivity: what is the connection? *Microorganisms* 7:374. 10.3390/microorganisms7100374 31547108 PMC6843312

[B25] Fu S. Guo S. Wang J. Wang Y. Zhang Z. Shen Z. (2018). Microbial community diversity of Jinghong laying hens at peak production based on 16S rRNA sequencing. *J. Appl. Anim. Res.* 46 1430–1436. 10.1080/09712119.2018.1520713

[B26] Han G. G. Kim E. B. Lee J. Lee J.-Y. Jin G. Park J. (2016). Relationship between the microbiota in different sections of the gastrointestinal tract, and the body weight of broiler chickens. *Springerplus* 5:911. 10.1186/s40064-016-2604-8 27386355 PMC4927549

[B27] Hubert S. M. Al-Ajeeli M. Bailey C. A. Athrey G. (2019). The role of housing environment and dietary protein source on the gut microbiota of chicken. *Animals* 9:1085. 10.3390/ani9121085 31817422 PMC6940977

[B28] Hy-Line. (2016). *Management Guide For Hy-Line Brown Laying Hen In Alternative Systems [Online].* UK. Available online at: https://www.hyline.com/userdocs/pages/B_ALT_COM_ENG.pdf (accessed 15 May, 2019).

[B29] Janczak A. M. Riber A. B. (2015). Review of rearing-related factors affecting the welfare of laying hens. *Poult. Sci.* 94 1454–1469. 10.3382/ps/pev123 26009752 PMC4991062

[B30] Joat N. Van T. T. H. Stanley D. Moore R. J. Chousalkar K. (2021). Temporal dynamics of gut microbiota in caged laying hens: a field observation from hatching to end of lay. *Appl. Microbiol. Biotechnol.* 105 4719–4730. 10.1007/s00253-021-11333-8 34014348

[B31] Jurburg S. D. Brouwer M. S. M. Ceccarelli D. van der Goot J. Jansman A. J. M. Bossers A. (2019). Patterns of community assembly in the developing chicken microbiome reveal rapid primary succession. *Microbiologyopen* 8:e00821. 10.1002/mbo3.821 30828985 PMC6741130

[B32] Klindworth A. Pruesse E. Schweer T. Peplies J. Quast C. Horn M. (2013). Evaluation of general 16S ribosomal RNA gene PCR primers for classical and next-generation sequencing-based diversity studies. *Nucleic Acids Res.* 41:e1. 10.1093/nar/gks808 22933715 PMC3592464

[B33] Kraimi N. Calandreau L. Zemb O. Germain K. Dupont C. Velge P. (2019a). Effects of gut microbiota transfer on emotional reactivity in Japanese quails (*Coturnix japonica*). *J. Exp. Biol.* 222:jeb202879. 10.1242/jeb.202879 30975742

[B34] Kraimi N. Dawkins M. Gebhardt-Henrich S. G. Velge P. Rychlik I. Volf J. (2019b). Influence of the microbiota-gut-brain axis on behavior and welfare in farm animals: a review. *Physiol. Behav.* 210:112658. 10.1016/j.physbeh.2019.112658 31430443

[B35] Madlala T. Okpeku M. Adeleke M. A. (2021). Understanding the interactions between Eimeria infection and gut microbiota, towards the control of chicken coccidiosis: a review. *Parasite (Paris, France)* 28:48. 10.1051/parasite/2021047 34076575 PMC8171251

[B36] Ngunjiri J. M. Taylor K. J. M. Abundo M. C. Jang H. Elaish M. Mahesh K. C. (2019). Farm stage, bird age, and body site dominantly affect the quantity, taxonomic composition, and dynamics of respiratory and gut microbiota of commercial layer chickens. *Appl. Environ. Microbiol.* 85:9 10.1128/AEM.03137-18 30824436 PMC6495750

[B37] Nordentoft S. Mølbak L. Bjerrum L. De Vylder J. Van Immerseel F. Pedersen K. (2011). The influence of the cage system and colonisation of *Salmonella* Enteritidis on the microbial gut flora of laying hens studied by T-RFLP and 454 pyrosequencing. *BMC Microbiol.* 11:187. 10.1186/1471-2180-11-187 21859465 PMC3188488

[B38] Pan D. Yu Z. (2014). Intestinal microbiome of poultry and its interaction with host and diet. *Gut Microbes* 5 108–119. 10.4161/gmic.26945 24256702 PMC4049927

[B39] Parois S. Calandreau L. Kraimi N. Gabriel I. Leterrier C. (2017). The influence of a probiotic supplementation on memory in quail suggests a role of gut microbiota on cognitive abilities in birds. *Behav. Brain Res.* 331 47–53. 10.1016/j.bbr.2017.05.022 28502731

[B40] Pourabedin M. Zhao X. (2015). Prebiotics and gut microbiota in chickens. *FEMS Microbiol. Lett.* 362:fnv122. 10.1093/femsle/fnv122 26208530

[B41] Primary Industries Standing Committee (2002). *Model Code Of Practice For The Welfare Of Animals: Domestic Poultry.* Collingwood, Vic: CSIRO Publishing.

[B42] Richardson A. J. Calder A. G. Stewart C. S. Smith A. (1989). Simultaneous determination of volatile and non-volatile acidic fermentation products of anaerobes by capillary gas chromatography. *Lett. Appl. Microbiol.* 9 5–8. 10.1111/j.1472-765x.1989.tb00278.x

[B43] Rufener C. Berezowski J. Maximiano Sousa F. Abreu Y. Asher L. Toscano M. J. (2018). Finding hens in a haystack: consistency of movement patterns within and across individual laying hens maintained in large groups. *Sci. Rep.* 8:12303. 10.1038/s41598-018-29962-x 30120253 PMC6098140

[B44] Ruhnke I. Normant C. Raj R. V. Suchodolski J. Campbell D. L. M. Kheravii S. K. (2018). “The impact of range use on caecal microbiota composition in free-range laying hens,” in *Proceedings of the 29th Annual Australian Poultry Science Symposium*, (Sydney, NSW: University of Sydney, Poultry Research Foundation).

[B45] Rychlik I. (2020). Composition and function of chicken gut microbiota. *Animals* 10:103. 10.3390/ani10010103 31936291 PMC7022619

[B46] Saladrigas-García M. D’Angelo M. Ko H. L. Traserra S. Nolis P. Ramayo-Caldas Y. (2021). Early socialization and environmental enrichment of lactating piglets affects the caecal microbiota and metabolomic response after weaning. *Sci. Rep.* 11:6113. 10.1038/s41598-021-85460-7 33731752 PMC7969613

[B47] Schreuder J. Velkers F. C. Bossers A. Bouwstra R. J. de Boer W. F. van Hooft P. (2021). Temporal dynamics of cloacal microbiota in adult laying chickens with and without access to an outdoor range. *Front. Microbiol.* 11:626713. 10.3389/fmicb.2020.626713 33584593 PMC7876281

[B48] Schreuder J. Velkers F. C. Bouwstra R. J. Beerens N. Stegeman J. A. de Boer W. F. (2020). An observational field study of the cloacal microbiota in adult laying hens with and without access to an outdoor range. *Anim. Microbiome* 2:28. 10.1186/s42523-020-00044-6 33499947 PMC7807755

[B49] Scrinis G. Parker C. Carey R. (2017). The caged chicken or the free-range egg? The regulatory and market dynamics of layer-hen welfare in the UK, Australia and the USA. *J. Agric. Environ. Ethics* 30 783–808. 10.1007/s10806-017-9699-y

[B50] Seidlerova Z. Kubasova T. Faldynova M. Crhanova M. Karasova D. Babak V. (2020). Environmental impact on differential composition of gut microbiota in indoor chickens in commercial production and outdoor, backyard chickens. *Microorganisms* 8:767. 10.3390/microorganisms8050767 32443788 PMC7285315

[B51] Torok V. Hughes R. Ophel-Keller K. Ali M. MacAlpine R. (2009). Influence of different litter materials on cecal microbiota colonization in broiler chickens. *Poult. Sci.* 88 2474–2481. 10.3382/ps.2008-00381 19903943

[B52] van der Eijk J. A. J. Rodenburg T. B. de Vries H. Kjaer J. B. Smidt H. Naguib M. (2020). Early-life microbiota transplantation affects behavioural responses, serotonin and immune characteristics in chicken lines divergently selected on feather pecking. *Sci. Rep.* 10:2750. 10.1038/s41598-020-59125-w 32066789 PMC7026165

[B53] van der Eijk J. A. de Vries H. Kjaer J. B. Naguib M. Kemp B. Smidt H. (2019). Differences in gut microbiota composition of laying hen lines divergently selected on feather pecking. *Poult. Sci.* 98 7009–7021. 10.3382/ps/pez336 31226709 PMC6869756

[B54] Videnska P. Sedlar K. Lukac M. Faldynova M. Gerzova L. Cejkova D. (2014). Succession and replacement of bacterial populations in the caecum of egg laying hens over their whole life. *PLoS One* 12:e115142. 10.1371/journal.pone.0115142 25501990 PMC4264878

[B55] Villageliu D. N. Lyte M. (2017). Microbial endocrinology: why the intersection of microbiology and neurobiology matters to poultry health. *Poult. Sci.* 96 2501–2508. 10.3382/ps/pex148 29050443

[B56] Waite D. W. Taylor M. W. (2014). Characterizing the avian gut microbiota: membership, driving influences, and potential function. *Front. Microbiol.* 5:223. 10.3389/fmicb.2014.00223 24904538 PMC4032936

[B57] Wang L. Lilburn M. Yu Z. (2016). Intestinal microbiota of broiler chickens as affected by litter management regimens. *Front. Microbiol.* 7:593. 10.3389/fmicb.2016.00593 27242676 PMC4870231

[B58] Wen C. van Dixhoorn I. Schokker D. Woelders H. Stockhofe-Zurwieden N. Rebel J. M. J. (2021). Environmentally enriched housing conditions affect pig welfare, immune system and gut microbiota in early life. *Anim. Microbiome* 3:52. 10.1186/s42523-021-00115-2 34321110 PMC8320228

[B59] Yan W. Sun C. Yuan J. Yang N. (2017). Gut metagenomic analysis reveals prominent roles of *Lactobacillus* and cecal microbiota in chicken feed efficiency. *Sci. Rep.* 7:45308. 10.1038/srep45308 28349946 PMC7365323

[B60] Yeoman C. J. Chia N. Jeraldo P. Sipos M. Goldenfeld N. D. White B. A. (2012). The microbiome of the chicken gastrointestinal tract. *Anim. Health Res. Rev.* 13:89. 10.1017/S1466252312000138 22853945

[B61] Zakrzewski M. Proietti C. Ellis J. J. Hasan S. Brion M.-J. Berger B. (2017). Calypso: a user-friendly web-server for mining and visualizing microbiome–environment interactions. *Bioinformatics* 33 782–783. 10.1093/bioinformatics/btw725 28025202 PMC5408814

[B62] Zhou J. Wu S. Qi G. Fu Y. Wang W. Zhang H. (2021). Dietary supplemental xylooligosaccharide modulates nutrient digestibility, intestinal morphology, and gut microbiota in laying hens. *Anim. Nutr.* 7 152–162. 10.1016/j.aninu.2020.05.010 33997343 PMC8110867

